# Activation of a TLR9 mediated innate immune response in preeclampsia

**DOI:** 10.1038/s41598-019-42551-w

**Published:** 2019-04-11

**Authors:** Rachel D. Williamson, Fergus P. McCarthy, Louise C. Kenny, Cathal M. McCarthy

**Affiliations:** 1 Irish Centre for Fetal and Neonatal Translational Research (INFANT), Cork University Maternity Hospital, Cork, Ireland; 2 Department of Pharmacology and Therapeutics, Western Gateway Building, University College Cork, Cork, Ireland; 3 Department of Women’s and Children’s Health Institute of Translational Medicine, University of Liverpool, Liverpool, United Kingdom

## Abstract

Preeclampsia is a multisystemic disorder leading to the development of a placental ischemic microenvironment with a resultant increase in oxidative stress. There is evidence that mitochondrial dysfunction and the innate immune system both play a role in the pathophysiology of this disease. Mitochondrial DAMPs such as mtDNA bind specific pattern recognition receptors such as Toll-like receptor 9 (TLR9) on the endosomal surface of immune cells, in particular neutrophils, subsequently activating them and triggering an innate response. We hypothesised that the exaggerated innate immune response seen in preeclampsia is provoked by dysfunctional mitochondria. Here we provide evidence that TLR9 activity is significantly increased at time of disease in women with preeclampsia. Furthermore, we show activation of neutrophil markers, Calprotectin, Myeloperoxidase (MPO), and IL-8 are significantly increased at time of disease compared to uncomplicated pregnancies. This research supports a potential role of TLR9 activation of an innate immune response evident in preeclampsia which may possibly be initially triggered by dysfunctional mitochondria.

## Introduction

Preeclampsia is multifactorial disorder of pregnancy that is defined by the onset of hypertension and proteinuria after 20 weeks’ gestation. One of the most established characteristics of this disorder is the inability of the trophoblasts to invade the maternal uterine decidual arteries, resulting in poor placentation. Production of placental anti-angiogenic factors such as soluble fms-related tyrosine kinase 1 (sFlt-1) and soluble endoglin (sEng) have been extensively researched in pre-eclampsia^1,2^. More recently, the cardiovascular system and its role in the development of preeclampsia is being explored^3^. Other pathological characteristics of preeclampsia include placental and systemic oxidative stress and dysfunction of the maternal vasculature^4,5^. In normal pregnancies there is evidence of a controlled systemic inflammatory response where cytokines promote the infiltration of the spiral arteries by invading trophoblast cells^6^. This controlled inflammatory response becomes dysregulated in preeclampsia resulting in abnormal activation of monocytes, neutrophils and the endothelium causing maternal inflammation^7^. There is overwhelming evidence that oxidative stress plays a key role in the pathophysiology of preeclampsia^8,9^. In turn oxidative stress, as a result of a placental ischemic microenvironment, releases reactive oxygen species into the maternal circulation, which can provoke a systemic inflammatory response^10^. The innate immune system acts as both a protector and effector during pregnancy. The innate system encompasses neutrophils, dendritic cells, natural killer cells and macrophages and these immune responders are activated to protect the mother from pathogens. Toll-like receptors (TLRs) are a family of type I transmembrane pattern recognition receptors (PRRs) that identify invading pathogens or endogenous damage signals and instigate an innate immune response. TLR9 can detect conserved sequences known as pathogen-associated molecular patterns (PAMPs) and also specifically respond to endogenous molecular structures known as damage-associated molecular patterns (DAMPs) via unmethylated CpG dinucleotide motifs as evident on mitochondrial DNA^11^. Activation of endosomal TLR9 involves an intracytoplasmic signalling cascade that leads to the up-regulation of pro-inflammatory transcription factors and subsequent liberation of pro-inflammatory cytokines^12^. There is evidence of increased TLR9 expression in both placental tissue and peripheral blood mononuclear cells (PBMC) from patients with preeclampsia compared with controls^13,14^.

Neutrophils are short-lived effector cells of the innate immune system. These immune cells are activated in women during pregnancy and are further stimulated in preeclampsia^15^. Histopathological evidence has shown extensive infiltration of neutrophils in the systemic vasculature of women with preeclampsia compared to controls^16,17^. Neutrophils express a number of TLR’s on their surface including TLR9^18^. Mitochondrial DNA (mtDNA) has been shown to activate neutrophils via a TLR9 signalling cascade, which elicits a neutrophil pro-inflammatory phenotype^19–21^. Neutrophil activation results in the secretion of a number of markers including reactive oxygen species (ROS), matrix metalloproteinase-8 (MMP-8), calprotectin, myeloperoxidase (MPO) and the pro-inflammatory cytokine IL-8^22^.

Our research has previously provided evidence of a role for mitochondrial dysfunction in the pathophysiology of preeclampsia with an increase in ΔmtDNA evident between 15 and 20 weeks in women with preeclampsia^23^. Interestingly we also showed a significant increase in mtDNA at time of disease (TOD) in women with preeclampsia compared to controls^24^. The aim of this study was to investigate if the increase in mtDNA we previously reported in preeclampsia triggers activation of TLR9 signalling cascade. We also wanted to determine if a subsequent neutrophil pro-inflammatory phenotype was elicited in preeclampsia compared to controls by measuring a number of neutrophil activation markers longitudinally in pregnancy. We hypothesised that dysfunctional mitochondria provokes an exaggerated innate immune response in preeclampsia.

## Results

### Determination of TLR9 activity and downstream markers of neutrophil activation at 15 weeks’ gestation

**Figure 1:**
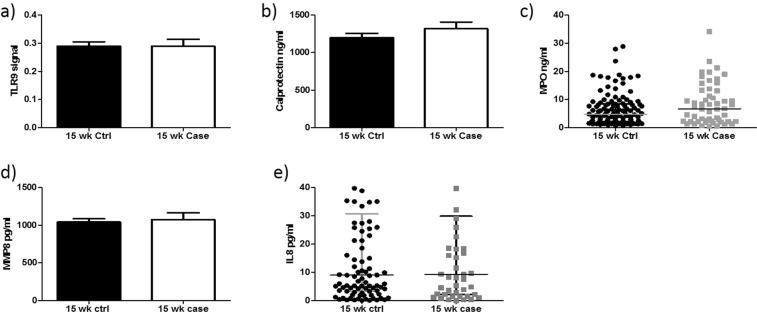
TLR9 activity and downstream markers of neutrophil activation did not alter at 15 weeks’ gestation. There was no significant increase in TLR9 activity (**a**) or neutrophil activation markers Calprotectin (**b**), MPO, (**c**) MMP-8 (**d**) and IL-8 (**e**) at 15 weeks’ gestation between cases and controls.

To determine if TLR9 was activated by circulating mediators present in preeclampsia, HEK-TLR9 cells were treated with 3% plasma. The level of neutrophil activation markers, calprotectin, MPO, MMP8 and IL-8 were also examined. Firstly, there was no significant increase in TLR9 activity at 15 weeks’ gestation in cases compared to controls (0.28 ± 0.02 v 0.2 ± 0.02; P = 0.99) (Fig. 1a). We subsequently measured a number of well described markers of neutrophil activation in both study groups at 15 weeks’ gestation. There was no significant increase in calprotectin (1319 ng/ml ± 87.96, v 1198 ng/ml ± 56.76; P = 0.23) (Fig. 1b) or myeloperoxidase (Median [IQR]: 6.72 ng/ml [2.20–13.13] v 4.83 ng/ml [2.11–8.41]; P = 0.14) between cases and controls (Fig. 1c). There was also no significant difference in MMP8 (1041.27 pg/ml ± 42.96 v 1075.62 pg/ml ± 88.24; P = 0.69) (Fig. 1d) or the pro-inflammatory cytokine IL-8 (median [IQR]: 8.99 [3.32–30.70] v 9.32 [2.18–29.85]; P = 0.95) between cases and controls at 15 weeks’ gestation (Fig. 1e).

### Determination of TLR9 activity and downstream markers of neutrophil activation at 20 weeks’ gestation

**Figure 2:**
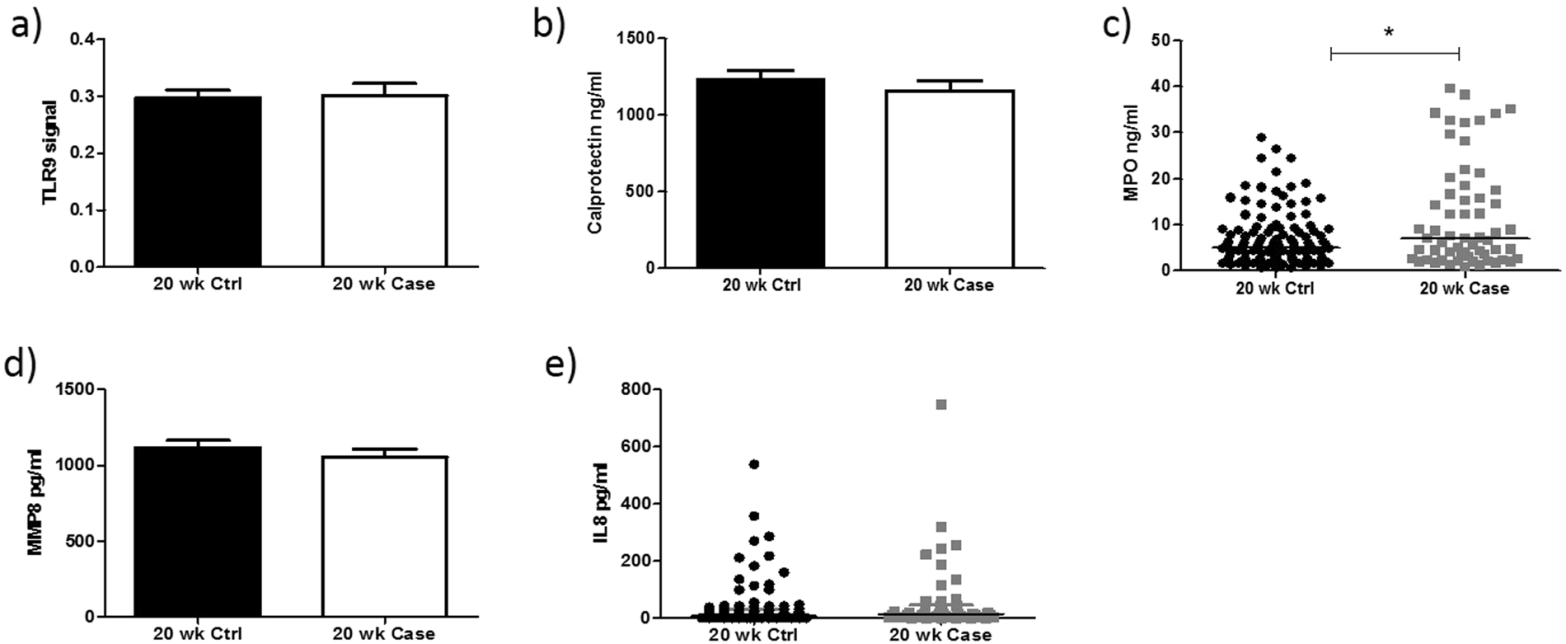
TLR9 activity and downstream markers of neutrophil activation did not alter at 20 weeks’ gestation. There was no significant increase in TLR9 activity (**a**) or neutrophil activation markers Calprotectin (**b**), MMP-8 (**d**) and IL-8 (**e**) at 20 weeks’ gestation between cases and controls. MPO expression (**c**) is significantly increased in cases compared with controls (P = 0.02).

There was no significant increase in TLR9 activity at 20 weeks’ gestation in cases compared to controls (0.29 ± 0.01, v 0.30 ± 0.02; P = 0.88) (Fig. 2a). While there was no change in the levels of calprotectin between cases and controls at 20 weeks’ gestation (1155 ng/ml ± 68.45 v 1234 ng/ml ± 61.10; P = 0.42) (Fig. 2b), there was a significant increase in myeloperoxidase in cases compared to controls (Median [IQR]: 5.02 ng/ml [2.36–9.08] v 7.07 ng/ml [2.74–17.24]; P = 0.02) (Fig. 2c). There was no significant change in the levels of MMP8 between cases and controls (1116.73 pg/ml ± 48.24 v 1053.74 pg/ml ± 54.05; P = 0.41) (Fig. 2d). There was no significant increase in IL-8 in cases compared to controls at 20 weeks’ gestation (Median [IQR]: 7.95 pg/ml [2.52–30.31] v 13.49 pg/ml [3.66–46.66]; P = 0.14) (Fig. 2e).

### Activation of TLR9 activity provokes a neutrophil-derived pro-inflammatory phenotype at time of disease in preeclampsia

**Figure 3:**
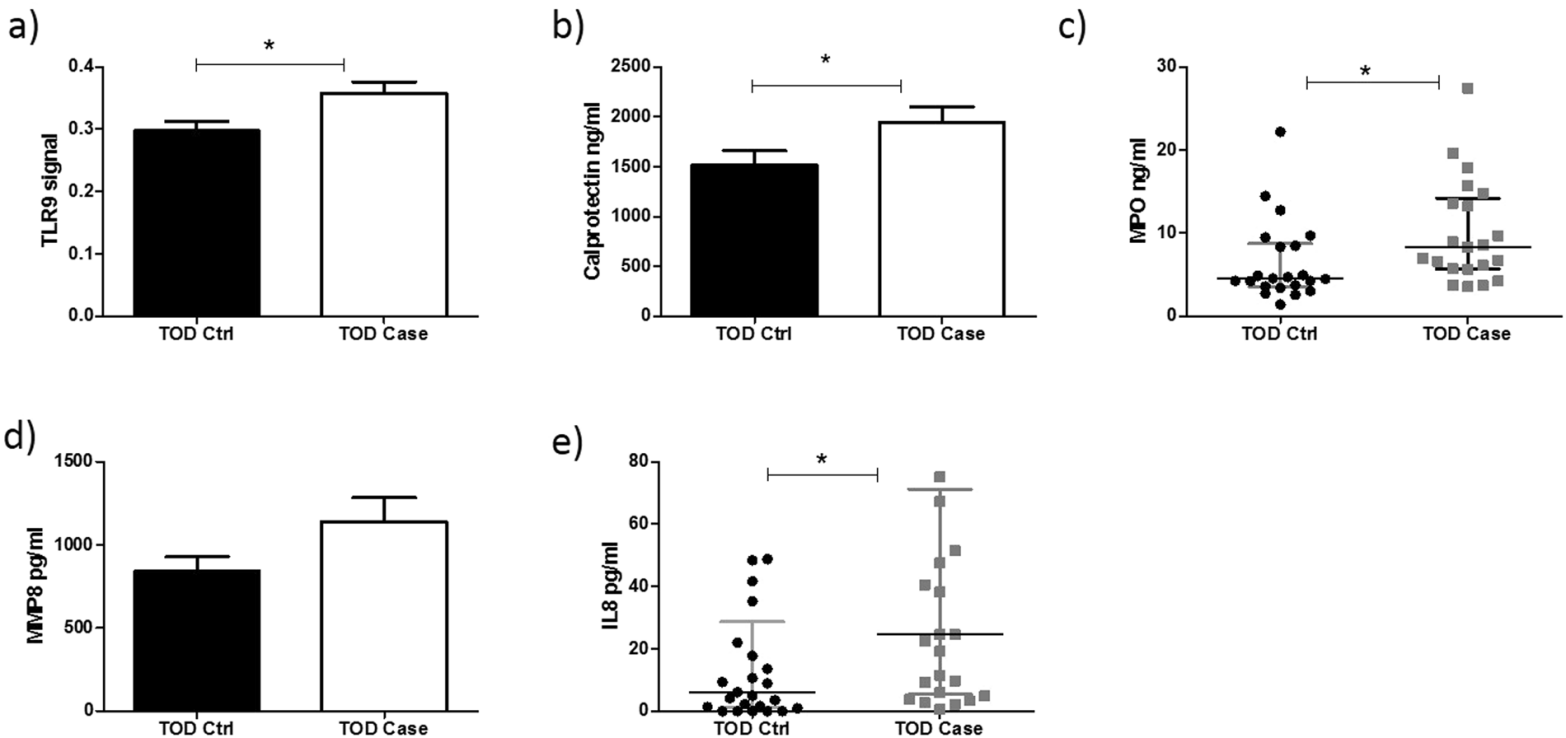
Circulating plasma mediators activate a TRL9-mediated innate immune response in preeclampsia at TOD. TLR-9 activity (**a**), Calprotectin (**b**), MPO (**c**), and IL-8 (**e**) are significantly increased at time of disease in cases compared to controls (P = 0.01). There was no significant increase in MMP-8 at TOD in cases in comparison to controls.

There was a significant increase in TLR9 activity at TOD in preeclampsia cases compared to controls (0.29 ± 0.01 v 0.35 ± 0.01; P = 0.01) (Fig. 3a). Subsequently, there was also a significant increase in both calprotectin (1946.55 ng/ml ± 155.08 v 1516.45 ng/ml ± 145.84; P = 0.04) (Fig. 3b) and myeloperoxidase (Median [IQR]: 8.33 ng/ml [5.70–14.20] v 4.52 ng/ml [3.53–8.73]; P = 0.01) (Fig. 3c) respectively at TOD in cases compared to controls. There was no significant increase in MMP-8 in cases when compared with controls at time of TOD, (842.92 pg/ml ± 87.07 v 1140.64 pg/ml ± 144.77; P = 0.08) (Fig. 3d). Finally, there was a significant increase in the pro-inflammatory cytokine IL-8 (Median [IQR]: 6.13 pg/ml [1.18–28.68] v 24.63 pg/ml [5.37–71.20]; P = 0.01) at TOD in cases compared to controls (Fig. 3e).

### Evidence of elevated innate immune response across gestation in preeclampsia

**Figure 4:**
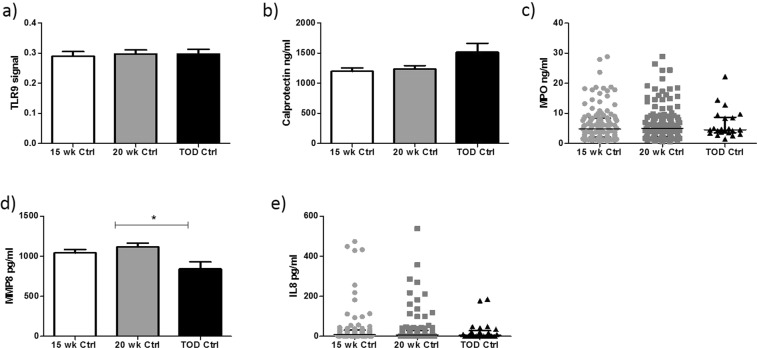
Normal pregnancy did not activate a TLR9 mediated innate immune response. There was no significant increase in TLR9 activity (**a**), Calprotectin (**b**), MPO (**c**) in healthy control pregnancies across gestation. MMP8 (**d**) is significantly reduced at term pregnancies (P = 0.01), while IL-8 (**e**) showed no significant difference across gestation in healthy control pregnancies.

We investigated if the neutrophil-mediated innate immune response were altered longitudinally in pregnancy. Initially, there was no difference in TLR9 activity across gestation in controls. (Fig. 4a). Similarly, there was no significant change in calprotectin, MPO and IL-8 expression in controls (Fig. 4b,c,e). MMP-8 was significantly decreased in uncomplicated pregnancies (Fig. 4d).

**Figure 5:**
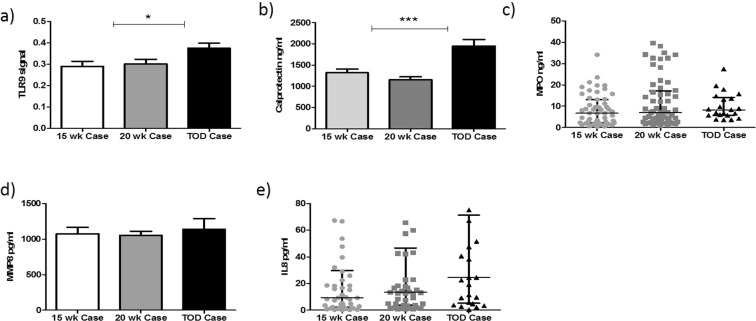
Neutrophil activation markers are increased across pregnancyin preeclampsia. TLR9 activity (**a**), Calprotectin (**b**), are both significantly increased across gestation in cases with preeclampsia (P = 0.01). There was no significant increase in neutrophil activation markers MPO (**c**), MMP8 (**d**) and IL-8 (**e**) across gestation in cases with preeclampsia.

In cases complicated with preeclampsia, both TLR9 and calprotectin were significantly increased across gestation (0.28 ± 0.02, 0.30 ± 0.02, 0.37 ± 0.02; P = 0.01) and (1318.69 ng/ml ± 87.95, 1233.77 ng/ml ± 68.45, 1946.55 ng/ml ± 145.84; P = 0.0001) respectively. (Fig. 5a,b). There was no significant increase in MPO, (6.72 ng/ml [2.20–13.13], 7.09 ng/ml [2.74–17.24], 8.33 ng/ml [5.70–14.20]; P = 0.14), MMP-8 (1075.62 pg/ml ± 88.24, 1053.74 pg/ml ± 54.05, 1140.64 pg/ml ± 87.08; P = 0.82) or IL-8 (9.32 pg/ml [2.18–29.85], 13.49 pg/ml [3.66–46.66], and 24.63 pg/ml [5.37–71.20]; P = 0.13) across gestation in preeclampsia cases (Fig. 5c–e).

### Determination of TLR9 activity and downstream markers of neutrophil activation in preterm cases versus term cases

We next explored if there was a difference in the neutrophil pro-inflammatory phenotype across all time-points in women who developed preeclampsia and delivered either before 37 weeks or after 37 weeks’ gestation (Table 1). There was no significant difference in TLR9 activity in preterm preeclampsia compared to term preeclampsia across gestation. Similarly, there was no statistical significance at each time-point when comparing expression of calprotectin, MPO and MMP8 in preterm preeclampsia versus term preeclampsia. Interestingly IL-8 expression is initially increased at 15 weeks’ gestation in women who developed preterm preeclampsia, but the cytokine levels reduce as pregnancy progresses.

**Table 1:** Plasma levels of TLR9 activity, neutrophil activation markers and pro-inflammatory cytokine IL-8 in patients with preterm and term preeclampsia.

Neutrophil markers	15 weeks (Preterm N = 19)	15 weeks (Term N = 38)	P Value	20 weeks (Preterm N = 19)	20 weeks (Term N = 37)	P Value	TOD (Preterm N = 6)	TOD (Term N = 16)	P Value
TLR9 Signal	0.31 ± 0.05	0.27 ± 0.03	0.66	0.30 ± 0.06	0.29 ± 0.03	0.41	0.37 ± 0.03	0.39 ± 0.04	0.713
Calprotectin (ng/ml)	1043.15 ng/ml ± 126.19	1645.66 ng/ml ± 187.30	0.16	886.99 ± ng/ml 245.48	1167.96 ng/ml ± 77.80	0.57	1878.35 ng/ml ± 424.90	2055.57 ng/ml ± 170.62	0.77
MPO (ng/ml)	4.42 ng/ml [1.77–10.25]	6.58 ng/ml [2.11–9.70]	0.56	4.55 ng/ml [2.48–8.74]	6.15 ng/ml [2.96–21.46]	0.84	8.59 ng/ml [4.72–11.87]	8.33 ng/ml [5.67–14.78]	0.43
MMP8 (pg/ml)	1138.41 pg/ml ± 149.24	989.38 pg/ml ± 113.31	0.25	1097.48 pg/ml ± 108.58	1098.46 pg/ml ± 88.69	0.97	1775.73 pg/ml ± 466.30	1263.75 pg/ml ± 182.92	0.12
IL-8 (pg/ml)	32.09 pg/ml [7.40–192.91]	8.12 pg/ml [2.19–18.25]	0.17	22.92 pg/ml [4.76–188.69]	12.67 pg/ml [2.11–18.23]	0.32	24.63 pg/ml [2.68–47.59]	24.63 pg/ml [9.78–75.14]	0.81

Data is presented as mean ± SEM and median (25–75 percentiles) where applicable.

## Discussion

Pregnancy is associated with a maternal systemic inflammatory response; however, this response is exaggerated in preeclampsia. There has been a number of studies which have provided evidence of increased neutrophil activation in women with preeclampsia^16,25,26^. In this present study, we investigated if circulating mediators (including mtDNA)^23^, trigger TLR9 activity with the subsequent downstream activation of a neutrophil-mediated innate immune response in women with preeclampsia and healthy controls. We showed TLR9 activity is significantly increased in women with preeclampsia compared to healthy controls at time of disease. There was a corresponding increase in production of downstream neutrophil activation markers, calprotectin, myeloperoxidase, MMP8 and the pro-inflammatory cytokine IL-8 in women with preeclampsia compared to healthy controls at time of disease, indicating that complete activation of neutrophil pro-inflammatory phenotype only became evident late in pregnancy in preeclampsia.

The innate immune response plays a well described role in the pathophysiology of preeclampsia. Mitochondrial DAMPs such as mtDNA bind specific pattern recognition receptors such as TLR9 on the endosomal surface of the immune cells, in particular, neutrophils and activate an innate response. TLR9 receptors are also localised to trophoblasts and the villous stromal vascular endothelium and their expression is increased in preeclampsia^13^. We have shown a significant increase in TLR9 activity using a reporter cell assay incubated with plasma taken at time of disease in preeclampsia cases compared to healthy controls. Furthermore, previous work in our lab using the same study group samples examined mtDNA (as a marker of mitochondrial dysfunction) and reported that mtDNA was also significantly increased at time of disease in preeclampsia cases in the same study cohort^23^. This indicates that the increase in mitochondrial DAMP (mtDNA) at time of disease may activate a TLR9 mediated innate immune response in preeclampsia cases only. Exciting new work by He *et al*. has identified an additional role for TLR9 in preeclampsia where they established that TLR9 supressed angiogenesis in part by increasing sFlt-1 expression in a murine model of preeclampsia^27^. Furthermore, research carried out in spontaneous hypertensive rats, illustrated circulating mtDNA may lead to the activation of the innate immune system through TLR9^21^. Research by Goulopoulou *et al*., has also shown preliminary data indicating that TLR9 activation provokes preeclampsia–like symptoms in pregnant rats^20^ emphasising the pathogenic role of TLR9 in preeclampsia.

Calprotectin is a calcium binding protein and is located in the cytosol of neutrophils and is released upon neutrophil activation. In our study, calprotectin was significantly increased at time of disease compared to controls. This is in agreement with recent studies which have reported increase in calprotectin at term in preeclampsia^26^. Akçum *et al*., reported increased circulating calprotectin levels at term in women with preeclampsia, and interestingly found an additional increase in women with severe preeclampsia.

MPO is a lysosomal enzyme mainly produced and released by activated neutrophils. In this study, we showed a significant increase in MPO in cases as early as 20 weeks’ gestation when compared with controls and this significant increase was equally evident at time of disease in preeclampsia. Previous studies measuring MPO in preeclampsia have reported conflicting results; some studies have reported no difference in MPO in preeclampsia in samples taken at 24 weeks or later in the third trimester^28,29^, whereas Gandley *et al*., reported a 3-fold increase in circulating MPO levels in women with preeclampsia compared to matched healthy controls at 32–38 weeks’ gestation^30^. The variation in MPO levels in previous preeclampsia studies may be due to small sample size. In our study we compared 60 cases and 120 matched controls, which to our knowledge is one of the biggest studies carried out in relation to MPO and preeclampsia.

MMP8 is part of the MMP family which consists of 23 zinc and calcium dependent proteases that effect different mechanisms of the extracellular matrix. In recent years, MMPs have become a target of interest in preeclampsia due to its role in vascular function and remodelling^31,32^. Recent research shows strong evidence that MMP8 play an important role in mediating endothelial cell angiogenesis^32^. Furthermore, endothelial dysfunction is a pathogenic characteristic of preeclampsia, dysregulated MMP8 expression may play a crucial role in the disruption of angiogenesis in preeclampsia leading to endothelial dysfunction. In our study, there was an increase in circulating levels of MMP8 expression at TOD in women with preeclampsia cases compared to healthy controls.

Cytokines and chemokines have been extensively studied as markers of inflammation involved in the pathophysiology of preeclampsia. In this study, we showed a significant increase in circulating IL-8 levels at time of disease in cases compared to controls. IL-8 has previously shown to be increased in women with preeclampsia in the third trimester^33^. A recent study reported a significant increase in IL-8 in early gestation (5–15 weeks’) in women who subsequently went on to develop preeclampsia. However, a limitation of their study was the small sample size (n = 9)^34^. Other studies investigating IL-8 throughout pregnancy reported similar results to ours with no significant increase evident in the second trimester^35,36^ but a significant increase in IL-8 is evident in the third trimester in women with preeclampsia compared to healthy controls^37^.

Preterm preeclampsia occurs before 37 weeks’ gestation and is frequently regarded as a slightly different phenotype to preeclampsia occurring at term. Therefore, we investigated whether neutrophil activation triggers an altered innate immune response in preterm preeclampsia compared to term preeclampsia. In this study, there was no statistically significant difference in the concentration of neutrophil activation makers in preterm versus term in our cohort.

When investigating the activation of a TLR9 mediated innate immune response across pregnancy, there was no significant difference in expression of any of the neutrophil markers measured in healthy controls. However, in preeclampsia, significant increases were seen in neutrophil activation markers, TLR9 and calprotectin, illustrating that the innate immune response may be initially triggered earlier in preeclampsia but complete activation is not significantly evident until later in pregnancy. This is in agreement with recent studies of maternal inflammation (cytokines such as IL-6, TNF-α) in preeclampsia, whereby low level inflammation is evident early in pregnancies but is amplified in the third trimester of pregnancy^38–40^. This current study shows that the TLR9 activation of the innate immune system may play a role in the pathophysiology of preeclampsia in late gestation.

## Conclusion

Here we provide evidence that circulating plasma mediators may activate a TRL9-mediated innate immune response in preeclampsia. We show that the activity of TLR9, a receptor for mtDNA, is significantly increased at time of disease in preeclampsia. Subsequently we have shown increased production of neutrophil activation markers particularly late in pregnancy in preeclampsia. Finally, we have shown that possible activation of TLR-9 by dysfunctional mitochondria may provoke an exaggerated neutrophil-mediated innate immune response in preeclampsia.

## Material and Methods

### Study subjects

Subjects were recruited from the Screening for Pregnancy Endpoints (SCOPE) study Ireland which is an international multicentre prospective cohort study of nulliparous singleton pregnancies aimed to develop a screening test to predict adverse pregnancy outcomes including preeclampsia, SGA infants and spontaneous pre-term birth^41,42^. A nested case-control study within SCOPE Ireland was conducted which included all preeclampsia cases in SCOPE Ireland and matched controls with a case-to-control ratio of 1:2. Preeclampsia cases was defined as a systolic blood pressure ≥140 mm Hg and/or diastolic blood pressure ≥90 mm Hg on at least two occasions 4 hrs apart after 20 weeks’ gestation and with proteinuria (24 hour urinary protein ≥300 mg or urine dipstick protein ≥+2). Randomly selected controls were taken from healthy pregnant women who had uncomplicated pregnancies which were defined as pregnancies not affected by preeclampsia, preterm birth or growth restriction and delivered at >37 weeks. All blood pressure readings were <140 and/or <90 mmHg prior to the onset of labour. These were matched with the cases for maternal age, body mass index (BMI) and gestational age. Both 15 and 20 week samples were taken from the SCOPE study from women who subsequently went onto develop preeclampsia (n = 60) and controls (n = 120). Samples were also taken from a subset of women (n = 25) at the time of disease (TOD) with preeclampsia and matched controls. The 60 women with preeclampsia were composed of 39 women who developed term preeclampsia and 21 preterm preeclampsia cases. The SCOPE study was conducted according to the guidelines laid down in the Declaration of Helsinki, and all the procedures were approved by the Clinical Research Ethics Committee of the Cork Teaching (EMC5(10)05/02/08), and all women provided written informed consent.

### Sample collection

Plasma samples were collected in BD Heparin Vacutainer tubes, placed on ice, and centrifuged at 2,400 g for 10 minutes at 4 °C according to a standardised protocol. Plasma samples were stored at −80 °C until analysis.

### TLR-9 activity

TLR9 ligand activity was monitored with HEK-blue TLR9 Reporter Cell assay (InvivoGen). All experiments were performed using a cell density of 50,000 cells in a 96 well plate. Cells were initially seeded and left overnight prior to treatment for 24 hrs with 3% plasma taken at 15 and 20 weeks’ gestation (cases, n = 60, controls, n = 120) and TOD (cases, n = 25, controls, n = 25). The supernatant was incubated with Quanti-Blue detection medium (InvivoGen) and the activity was read on a Varioskan Flash plate reader (Thermo Scientific) at 630 nm.

### Calprotectin analysis

Plasma calprotectin (S100A8/S100A9) concentrations were measured by enzyme-linked immunosorbent assay (ELISA) using human S100A8/S100A9 Quantikine kit. Heparin plasma samples from all time-points were initially diluted a 100-fold in assay buffer and then directly added to a pre-coated plate. The ELISA was performed as per manufacturers’ instructions.

### Myeloperoxidase, MMP8, IL-8 analysis

Myeloperoxidase (MPO), Matrix metalloproteinases-8 (MMP8) and interleukin-8 (IL-8) concentrations were measured respectively at all time-points by individual ELISA DuoSet kits (R&D SYSTEMS. USA & Canada). ELISA was carried out as per manufacturer’s instructions.

### Statistical analysis

Analysis was performed using GraphPad Prism. Data were presented using median (±Interquartile range [IQR]) and comparisons of data between cases and controls were performed using a non-parametric Mann Whitney U test or Wilcoxon signed rank test as appropriate when data was not normally distributed. Data that was normally distributed were represented as mean (±SEM) and comparisons of data between cases and controls were performed using an unpaired t-test. P values < 0.05 were considered as statistically significant.
